# AdipoR1/APPL1 Potentiates the Protective Effects of Globular Adiponectin on Angiotensin II-Induced Cardiac Hypertrophy and Fibrosis in Neonatal Rat Atrial Myocytes and Fibroblasts

**DOI:** 10.1371/journal.pone.0103793

**Published:** 2014-08-06

**Authors:** Tengwei Cao, Zhen Gao, Lingyun Gu, Minglong Chen, Bing Yang, Kejiang Cao, He Huang, Mingfang Li

**Affiliations:** 1 College of Biotechnology and Pharmaceutical Engineering, Nanjing Tech University, Nanjing, P.R. China; 2 Division of Cardiology, Jiangyin Hospital Affiliated to Southeast University, Jiangyin, P.R. China; 3 Division of Cardiology, Department of Medicine, The First Affiliated Hospital of Nanjing Medical University, Nanjing, P.R. China; 4 State Key Laboratory of Materials-Oriented Chemical Engineering, Nanjing, P.R. China; University of Western Ontario, Canada

## Abstract

Atrial hypertrophy and fibrosis are essential pathological features of atrial fibrillation. Recently, adiponectin has become a protein of interest due to its beneficial effects on cardiovascular diseases. However, the molecular mechanism of atrial structural remodeling and signaling pathways evoked by adiponectin remain unclear. In the present study, we investigated the cardioprotective effect of globular adiponectin (gAcrp) on angiotensin II-induced atrial hypertrophy and fibrosis in neonatal Sprague-Dawley rat. To further investigate the molecular mechanisms underlying the preventive effect of gAcrp, transfection of cells with siRNA was used to suppress the mRNA expression of adiponectin receptor 1 (AdipoR1) and its downstream adaptor protein APPL1. Non-silencing-Cy-3 labelled siRNA was used to determine transfection efficiency using fluorescence microscopy. The expression of atrial natriuretic peptide and procollagen type1 α-1, hypertrophy marker and fibrosis one, respectively, was detected by real-time PCR. Furthermore, the expression of adenosine monophosphate-activated protein kinase (AMPK), phosphatidylinositol 3-kinase (PI3K) and Akt was detected by western blotting. In addition, nuclear p65 translocation activity was analyzed by EMSA supershift assay. Our results showed that AdipoR1 and the adaptor protein APPL1 mediated the protective effects of gAcrp. In addition, the function of adiponectin and phosphorylation of AMPK were prominently diminished by inhibition of PI3K. Furthermore, nuclear factor-κB (NF-κB) transcription was diminished by the specific inhibition of AMPK. Taken together, AMPK pivotally interacts with NF-κB and PI3K, mediating the cardioprotective effect of adiponectin, and may serve as a therapeutic target for preventing atrial hypertrophy and fibrosis. Our present study suggests that gAcrp could ameliorate AngII-induced cardiac hypertrophy and fibrosis in rat atrial cells, which is mediated by the activation of AMPK signaling pathways. APPL1 and AdipoR1 are the key factors involved in the downstream of gAcrp approach.

## Introduction

Atrial fibrillation (AF) is a common sustainable arrhythmia in clinical practice [1]. AF is one of the leading causes of stroke among the elderly and accounts for one-third of strokes among patients over the age of 65 [2]. Atrial structural remodeling is one of the most pivotal substrates in AF and leads to progressive architectural aggravation of atria after continuous episodes of AF. The primary changes of structural remodeling involve myocytic hypertrophy, myolysis, and interstitial fibrosis [3]. It has been demonstrated that the activation of the renin-angiotensin system (RAS) plays a pivotal role in atrial structural remodeling of AF [4], [5]. In addition, angiotensin II (AngII) has been shown to be a key trigger of atrial hypertrophy and fibrosis. However, the molecular mechanism of atrial structural remodeling still remains unclear.

At the cellular level, hypertrophy of cardiac ventricles is a result of increased cardiomyocyte cell volume, which is a procedure resulting from cellular signaling cascades and modulation of cellular energy mobilization [6]. One frequent observation of hypertrophy involves the fetal programming of gene expression with atrial natriuretic peptide (ANP) in ventricular myocytes and procollagen type1 α-1 (COL1A1) in ventricular fibroblasts.

Adiponectin (also known as Acrp30, AdipoQ, and GBP28), an adiocytokine secreted by adipocytes, has been the recent focus of intense research because of its insulin-sensitizing effect and possible therapeutic target for metabolic disorders [7], [8]. There is mounting evidence supporting that exogenous adiponectin plays a central modulatory role in various pathophysiological conditions. In addition to its well-characterized function in glucose and fatty acid metabolism, adiponectin has been extensively studied in recent years due to an apparent protective function in inflammation, metabolic syndrome, insulin resistance, atherosclerosis and cardiovascular disease [9]. The cardioprotective properties of adiponectin has been established recently in obesity-related diseases, including hypertrophic cardiomyopathy, myocardial ischemia–reperfusion injury, and heart failure [10]. Several studies have demonstrated an anti-hypertrophic effect of adiponectin on heart. Subsequently, these studies have also demonstrated that adiponectin influences cardiac remodeling in pathological conditions. Through stimulation of AMP-activated protein kinase signaling, adiponectin has been demonstrated to modulate the angiogenesis process *in* *vivo* in a mouse model of ischemia-induced angiogenesis [11]. It was previously reported that globular adiponectin inhibits AngII-induced cardiac ventricular hypertrophy [12]. Although many studies have attempted to elucidate the mechanisms responsible for cardiac ventricle remodeling, the various signaling pathways in atrial tissue remain unclear.

There are two putative distinct adiponectin receptors, AdipoR1 and AdipoR2, which are expressed at detectable levels in most mammalian tissues and cells. AdipoR1 is the preferentially abundant receptor expressed in skeletal muscle and heart tissues, and can be particularly detected in atrial myocytes [13]. The downstream adaptor protein, APPL1 (adaptor protein containing pleckstrin homology domain, phosphotyrosine binding domain and leucine zipper motif), interacts with AdipoR1. As such, both AdipoR1 and APPL1 participate in adiponectin-dependent activation of AMPK [14]. Despite detailed explanations in recent reports, both adiponectin and AMPK signaling pathways, and their regulation remain unclear in neonatal rat atrial myocytes and fibroblasts.

In the present study, we aimed to investigate the mechanism underlying the effect of globular adiponectin (gAcrp) on AngII-induced atrial impairment.

## Materials and Methods

### Materials

Recombinant rat globular adiponectin (gAcrp) was purchased from BioVision (Mountain View, CA). Adiponectin was produced using bacteria (*Escherichia coli*). Dulbecco’s modified eagle medium (DMEM), Trypsin-EDTA, collagenase, penicillin/streptomycin, Opti-MEM Reduced-Serum Medium and fetal bovine serum (FBS) were all obtained from Gibco Laboratories (Grand Island, NY). Antibodies for phosphor-AMPK, whole AMPK, phosphor-Akt, whole Akt, phosphor-STAT3, whole STAT3, APPL1, and NF-κB p65 were acquired from Cell Signaling Technology (Beverly, MA). The antibody for AdipoR1 was from Abcam (Cambridge, UK). All target siRNAs and Cy3-labeled non-silencing siRNA (NS siRNA) were purchased from Ribobio Co. (Guangzhou, China), and TransIT-TKO transfection reagent was acquired from Mirus Bio Corporation (Madison, WI). AngII, compound C, BrdU and LY294002 were acquired from Sigma-Aldrich (St. Louis, MO). Finally, Ro31-8220 was obtained from Millipore (Billerica, MA). All reagents were of analytical grade.

### Primary culture and identification of neonatal rat atrial myocytes and fibroblasts

All experimental procedures were approved by the Ethics Committee of Animal Research, Nanjing Medical University. Animals were used in agreement with the Animal Care and Experiment Committee of Nanjing Medical University guidelines. All primary cell extracts were performed under sodium pentobarbital anesthesia, and all efforts were made to minimize suffering. Neonatal rat atrial myocytes and fibroblasts were isolated as previously described [15]. In brief, the atria of 1 to 3-day-old Sprague-Dawley rats were minced under a dissecting microscope and dissociated in phosphate-buffered saline (PBS) containing 0.125% trypsin-EDTA and 0.05% type II collagenase for 6–8 cycles. Then, the cells were collected and incubated for 90 min at 37°C, 5% CO_2_ to allow the fibroblasts to adhere to tissue culture plates in high glucose (4.5 mg/l) DMEM with 10% FBS and 1% penicillin/streptomycin. The unattached myocytes were centrifuged and suspended in the same medium with 100 µM BrdU added. After 72 h, the atrial fibroblasts were removed using 0.125% trypsin-EDTA solution and passaged at a 1∶2 dilution. The second generation of fibroblasts were used in all subsequent experiments. The cells were serum starved for 12 h prior to commencing experiment. Of note, morphological examination and α-actin immunofluorescence were used to identify myocytes and ensure the positive rate of atrial myocytes approached 90%.

### mRNA expression analysis

To measure the mRNA levels of target genes, total RNA was isolated using Trizol reagent (Invitrogen) according to the manufacturer’s instructions. The RNA samples (500 ng per sample) were reverse transcribed to cDNA with a Transcriptor First Stand cDNA Synthesis Kit (Roche, Mannheim, Germany). Two-step real-time quantitative PCR (qPCR) was used to detect the mRNA expression levels with Power SYBR Green (Applied Biosystems, Warrington, UK). The qPCR was performed using Mastercycler Realplex^2^ (Eppendorf, Germany) with the following conditions: 95°C for preheating for 10 min, and then 40 cycles of 95°C for 15 sec and 60°C for 1 min. Serial dilutions (10-fold) of an external standard with a known concentration were used to create a standard curve for each primer pair. The qPCR primers are listed in Table 1. To confirm amplification specificity, each qPCR product was tested by melting curve analysis. The qPCR products were electrophoresed on a 1.0% agarose gel and stained with Gold View. The bands were visualized with the Molecular Imager ChemiDoc™ XRS + Imaging System (Bio-Rad, Hercules, CA). The relative quantification of gene expression was used for the determination of the expression of mRNA of interest in comparison to housekeeping gene GAPDH transcripts by the 2^−ΔΔCt^ method.

**Table 1:** Sequence of sense siRNA and QPCR primers.

Gene names	Sequences
*siRNA*	
APPL1	CUCACCUGACUUCGAAACUtt
AdipoR1	GAUGGAGGAGUUCGUAUAUtt
*qPCR primers*	
APPL1	F: 5′-CGTCCAGGAGGACAATCTCG-3′
	R: 5′-GACCAAAGGCTTTTGCCTGG-3′
AdipoR1	F: 5′-GCAGACAAGAGCAGGAGTGT-3′
	R: 5′-ACTGTGGTGGCCTTGACAAA-3′
ANP	F: 5′-CCGGTACCGAAGATAACAGC-3′
	R: 5′-CTCCAGGAGGGTATTCACCA-3′
COL1A1	F: 5′-GAGCGGAGAGTACTGGATCG-3′
	R: 5′-GTTCGGGCTGATGTACCAGT-3′
GAPDH	F: 5′-TCACCACCATGGAGAAGGC-3′
	R: 5′-GCTAAGCAGTTGGTGGTGCA-3′

### Western blot analysis

Atrial myocytes and fibroblasts grown on 6-well plates were harvested with 100 µl cell lysis buffer containingphosphatase inhibitor cocktail tablets (Roche, Mannhein, Germany) and phenylmethanesulfonyl fluoride (PMSF). Cells were scraped from the dish with a cell wiper to microfuge tubes. To maximize protein recovery, cells were also untrasonicated (Sonics & Materials, Newtown, CT). Protein concentrations in cell lysates were measured with a Pierce BCA protein assay kit (Thermo Scientific, Rockford, US). Protein samples (30 µg/lane) were separated by SDS-PAGE gel electrophoresis and transferred onto a 0.45-µm PVDF membranes (Millipore). The blots were blocked with 5% bull serum albumin for 1 h at room temperature and then probed with rabbit anti-rat antibody overnight at 4°C followed by horseradish peroxidase (HRP) conjugated goat anti-rabbit IgG secondary antibodies at room temperature for 2 h. The immunoreactive proteins were rinsed 3 times in TBST and visualized by enhanced chemiluminescence detection (ECL, Thermo Scientific). The band density was scanned and quantified by Image Lab 2.0 software (Bio-Rad). β-actin was used as a loading control for all samples.

### siRNA transfection and efficiency detection

Several 21-nucleotide small interfering RNA (siRNA) designed to knock down rat APPL1 and AdipoR1 were tested in neonatal atrial myocytes and fibroblasts. As it is difficult to transfect primary cells, we used TransIT-TKO, a broad spectrum siRNA transfection reagent that enables high efficiency of siRNA delivery and knockdown of target gene expression in many primary cells. As such 100 nM siRNA was transfected into the cells after incubation for 1 h in serum-free medium. After 24 h of incubation, the medium was replaced by serum-deprived medium, and the cells were treated with gAcrp. All transfections were performed according to manufacturers’ instructions and analyzed after 24 or 48 h of transfection. The transfection efficiency was determined using Cy3-tagged NS siRNA under a fluorescence microscope (Nikon) with excitation at 600 nm. All siRNA sequences are listed in Table 1.

### Nuclear and cytoplasmic protein extracts

Nuclear and cytoplasm extracts of neonatal rat atrial myocyte were prepared following the instructions of the relevant kits (Beyotime Institute of Biotechnology, Jiangsu, China). To determine NF-κB DNA binding ability, the nonisotopic Electrophoretic Mobility Shift Assay (EMSA) method with a Light Shift chemiluminescent EMSA kit (Pierce Biotechnology, Rockford, USA) was used. Briefly, nuclear extracts were incubated with biotin-labeled probes with the sequence 5′-AGT TGA GGG GAC TTT CCC AGG C-3′. The specificity of AngII-induced NF-κB activation was ascertained by supershift and competition experiments. Supershift groups were mixed with special anti-p65 antibody (1 µg), whereas the control was IgG (4 µg). After binding, the DNA-protein complexes were subjected to 6% native PAGE and transferred to a nylon membrane at 300 mA for 30 min and then cross-linked using ultraviolet rays for 20 min before detection by ECL chemiluminescence.

### Immunofluorescence staining

The expression of α-cardiac actin in atrial myocytes was evaluated using fluorescent staining. Cells cultured on coverslips were fixed with 4% paraformaldehyde for 15 min and then permeabilized in 0.5% Triton X-100 for 5 min at room temperature. After serial syringe dispensing of PBS, the coverslips were blocked in 5% bull serum albumin for 1 h. The primary antibodies (1∶50) were applied to the coverslips, and incubated overnight at 4°C. On The next day, the coverslips were washed 3 times again and incubated with secondary donkey anti-rabbit IgM-FITC antibody (Santa Cruz) for 1 h. After the nucleus was stained with DAPI, the coverslips were scanned under a fluorescence microscope. The microscopy images were visualized with ImageJ software.

### Statistical analysis

All data are presented as mean ± SD with the number of replicates (*n*) indicated in each case. Comparison of data between three or more groups was performed by one-way analysis of variance (ANOVA) with student-Newman-Keuls post hoc analysis using SPSS. Differences between groups were considered to be significant if the *p* value was <0.05.

## Results

### Globular adiponectin attenuates cardiac hypertrophy and fibrosis induced by AngII in myocytes and fibroblasts isolated from the atria of neonatal rats

**Figure 1:**
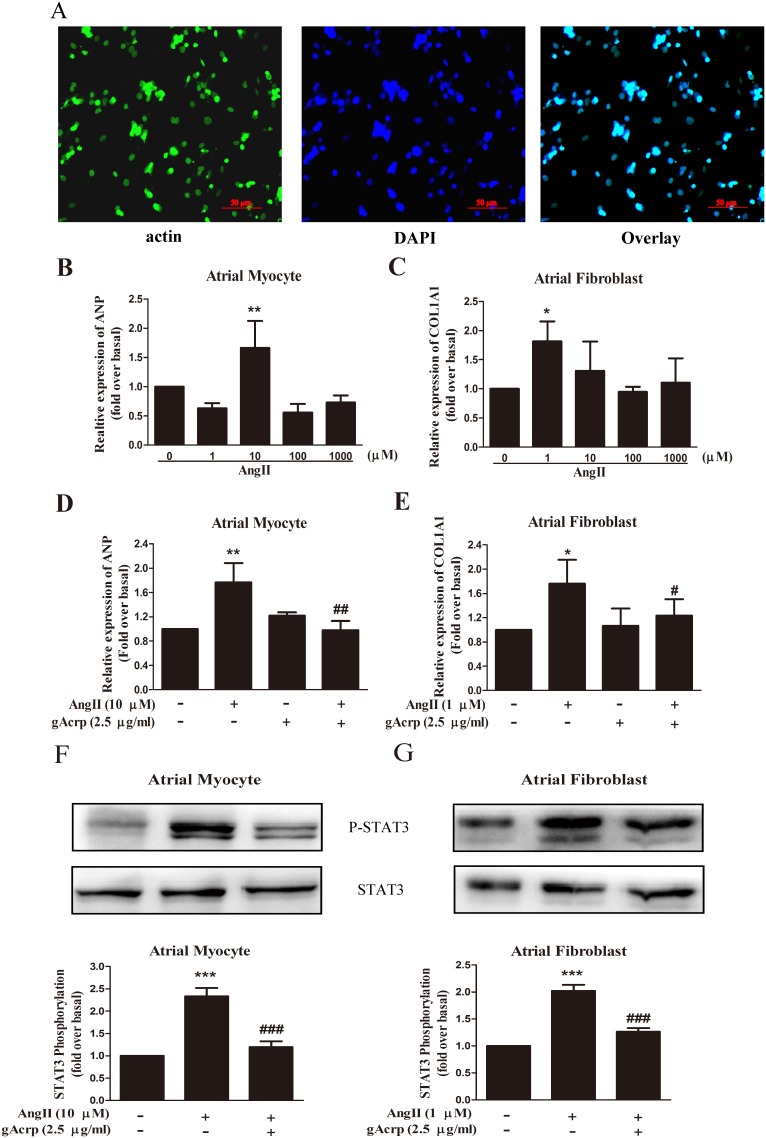
The effect of gAcrp on hypertrophy and fibrosis in atrial myocytes and fibroblasts treated with AngII. (A) Fluorescence microscopy images depict the α-cardiac actin (green) expression in atrial myocytes. The cell nuclei were stained with DAPI (blue). Scale bar, 50 µm. (B) and (C) Quantitative real time PCR analysis of ANP and COL1A1 expression in cultured atrial myocytes and fibroblasts treated with AngII (24 h). Data were expressed as mean ± SD, n = 3. *P<0.05, **P<0.01 vs. blank control. (D) and (E) Quantitative real time PCR analysis of AngII-stimulated expression of ANP and COLIA1 for 24 h after pretreatment with gAcrp (2.5 µg/ml) for 60 min in cultured atrial myocytes and fibroblasts. Data were expressed as mean ± SD, n = 3. *P<0.05, **P<0.01 vs. blank control; ^#^ *P*<0.05, ^##^ *P*<0.01 vs. AngII treatment. (F) and (G) Phosphorylation expression of STAT3 induced by AngII and attenuated by gAcrp (2.5 µg/ml) in cultured atrial myocytes and fibroblasts. Upper panels show immunoblotting representatively; bottom photos show quantification by densitometry. Whole cell lysates were subjected to Western blot analysis using a specific antibody as described in Materials and Methods. Data were expressed as mean ± SD of three independent experiments. ***P<0.001 vs. blank control; ^###^ *P*<0.001 vs. AngII infusion.

To mimic atrial fibrillation, we constructed a model of atrial hypertrophy and fibrosis with myocytes and fibroblasts isolated from neonatal SD rats. After 72 h of culture, the isolated myocytes and fibroblasts were attached to plates at the appropriate density (Figure. S1). The myocytes were identified using anti-α-actin antibody, which was recommended for the detection of rat α-cardiac actin [16]. As shown in Fig. 1A, atrial myocytes accounted for a large proportion of adherent cells. The results in Fig. 1B and C show that addition of AngII significantly increased ANP and COL1A1 expression in a dose-dependent manner. Nevertheless, concentration of AngII for the maximal effect varied in different cells, from 10 µM in atrial myocytes (*p<*0.01) down to 1 µM in atrial fibroblasts (*p*<0.05). All subsequent experiments were performed using AngII at these empirically determined concentration. After 24 h of culture in serum-deprived medium, both atrial myocytes and fibroblasts were pretreated with gAcrp (2.5 µg/ml) for 60 min prior to stimulation with AngII (Fig. 1D and E). AngII induced a significant increase in ANP mRNA expression by 76% (*p<*0.01) in atrial myocytes and COL1A1 mRNA expression by 72% (*p<*0.05) in atrial fibroblasts. However, the increase was inhibited by 45% (*p<*0.01) and 28% (*p<*0.05) by the pretreatment of gAcrp. We further examined the level of STAT3 phosphorylation after incubation with AngII (Fig. 1F and G). Globular adiponectin induced 55% reduction in STAT3 phosphorylation in atrial myocytes and 40% reduction in atrial fibroblasts (both *p<*0.001).

### Both APPL1 and AdipoR1 contribute to globular adiponectin-activated downstream signaling pathways

**Figure 2:**
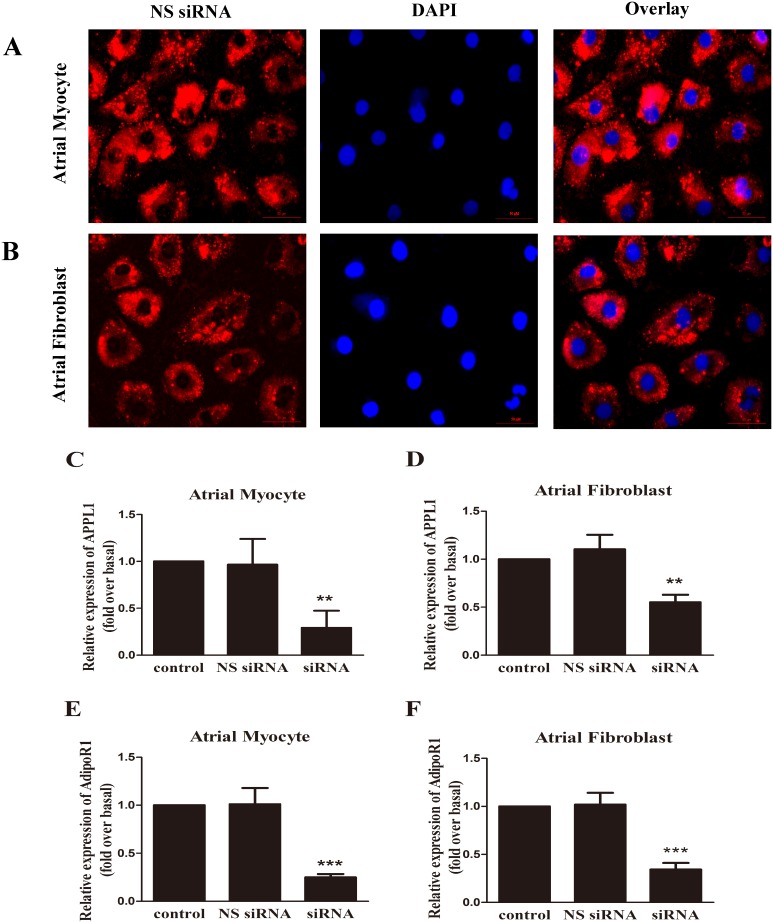
mRNA expression of APPL1 and AdipoR1 after transfection by specific siRNA in neonatal primary rat atrial myocytes and fibroblasts. (A) and (B) Fluorescence microscopy images showing the localization of the Cy3-tagged siRNA transfected atrial myocytes and fibroblasts for 24 h. The cell nuclei were stained with DAPI (blue). Scale bar, 50 µm. (C)–(F) Quantitative real time PCR analysis of the mRNA expression of APPL1 and AdipoR1 which was normalized by non-silencing (NS) siRNA. The concentration of siRNA was 100 nM appropriately. Data were expressed as mean ± SD of three independent experiments. **P<0.01; ***P<0.001 vs. nontransfected control.

**Figure 3:**
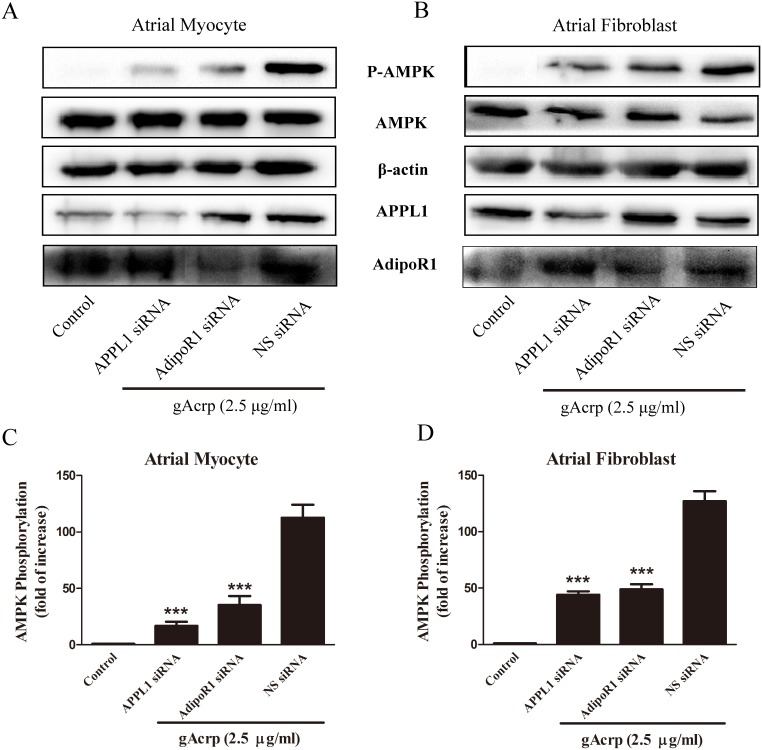
Involvement of APPL1 and AdipoR1 in AMPK activation after treatment with gAcrp in cultured atrial myocytes and fibroblasts. (A) After atrial myocytes were incubated with APPL1, AdipoR1 and non-silencing (NS) siRNA for 24 h, interference group treated with gAcrp (2.5 µg/ml) for 1 h. The expression of AMPK, pAMPK and APPL1 was determined by Western blotting. β-actin was used as an internal control. (B) After atrial fibroblasts were incubated with APPL1, AdipoR1 and non-silencing (NS) siRNA for 24 h, all the groups treated with gAcrp (2 µg/ml) for 1 h. (C) and (D) Quantitative analysis of AMPK phosphorylation shown in the upper panel was performed by densitometric analysis. Data were expressed as mean ± SD of three independent experiments. ***P<0.001 vs. NS siRNA control with gAcrp.

To further investigate the molecular mechanisms underlying the preventive effect of gAcrp on atrial hypertrophy and fibrosis, we examined whether APPL1 and AdipoR1 contributed to the downstream signaling pathways activated by gAcrp. We used siRNA transfection to suppress the mRNA expression of APPL1 and AdipoR1. As primary cultured cells can be difficult to be transfected with siRNA, we used Cy3-labeled NS siRNA to determine the transfection efficiency (Fig. 2A and B). The specific knockdown of APPL1 and AdipoR1 was verified by qPCR analysis (Fig. 2C, D, E and F). As shown in Fig. 2C and E, treatment with targeted siRNA dramatically reduced APPL1 mRNA expression by 70% (*p*<0.01) and AdipoR1 by 75% (*p*<0.001) in atrial myocytes. Similar to atrial myocytes, APPL1 and AdipoR1 expression were significantly decreased by treatment with targeted siRNA as compared with controls in atrial fibroblasts (Fig. 2D and F). In contrast, NS siRNA had no significant effect on APPL1 and AdipoR1 expression in both atrial myocytes and fibroblasts. After transfection with target siRNA, atrial myocytes and fibroblasts were incubated with gAcrp (2.5 µg/ml) for 1 hour. As illustrated in Fig. 3C and D, the phosphorylation of AMPK decreased by reducing APPL1 and AdipoR1 expression in atrial myocytes and fibroblasts. In contrast, the NS siRNA controls maintained a normal level of AMPK phosphorylation following gAcrp treatment.

**Figure 4:**
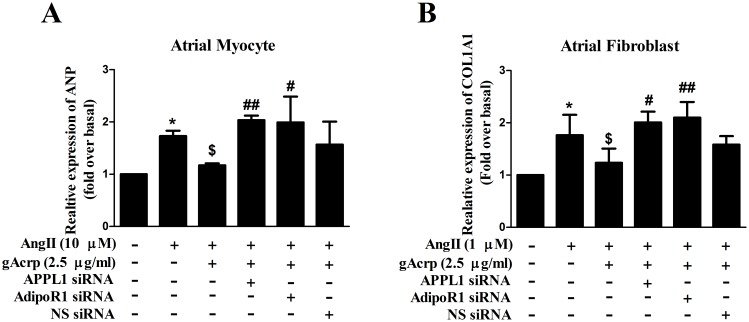
ANP and COL1A1 expression in atrial cells after RNA interference of APPL1 and AdipoR1. Atrial myocytes (A) and fibroblasts (B) were transfected with siRNA targeting APPL1 or AdipoR1, or non-silencing (NS) siRNA, respectively. After 24 h incubation, cells were pretreated with gAcrp (2.5 µg/ml) before stimulation with indicated concentration of AngII for 24 h. Relative expression level of ANP and COL1A1 was then measured as described previously. Data shown were expressed as mean ± SD of three independent experiments as. *P<0.05 vs. blank control, ^$^ *P*<0.05 vs. AngII infusion, ^#^ *P*<0.05; ^##^ *P*<0.01 vs. AngII + gAcrp infusion.

We further examined the functional significance of AdipoR1 and APPL1 interaction with gAcrp in AngII-treated atrial myocytes and fibroblasts. As shown in Fig. 4, real-time qPCR analysis indicated that ANP and COL1A1 mRNA expression increased after silencing of APPL1 or AdipoR1. The silencing of APPL1 and AdipoR1 significantly abrogated the protective effect of gAcrp, which suggests that APPL1 and AdipoR1 may play an important role in the interaction of downstream signaling pathways.

### Globular adiponectin mediates AMPK and PI3K/Akt signaling axis to protect atrial cells from hypertrophy and fibrosis

**Figure 5:**
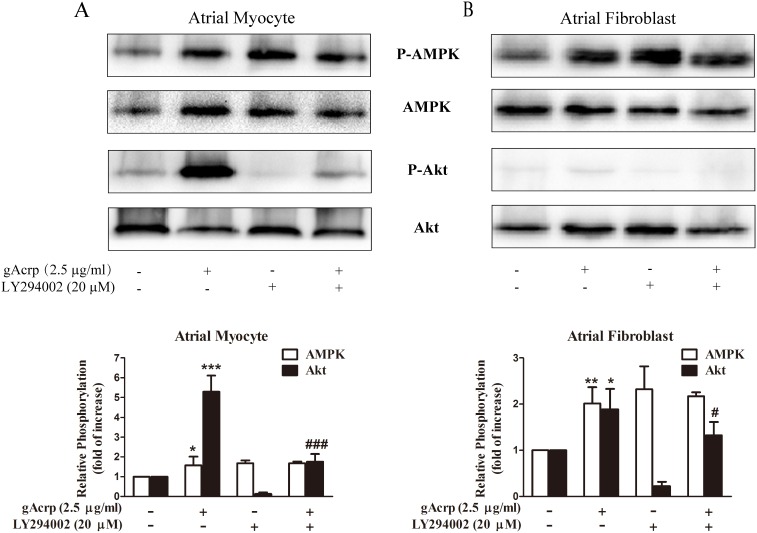
Role of PI3K in the activation of AMPK/Akt signaling pathway induced by gAcrp in atrial myocytes and fibroblasts. Cells were pretreated with 20 µM LY294002 for 1 h and then incubated with gAcrp (2.5 µg/ml) for another 1 h. Data were expressed as mean ± SD of three independent experiments. *P<0.05; **P<0.01; ***P<0.001 vs. blank control, ^#^ *P*<0.05; ^###^ *P*<0.001 vs. gAcrp only.

**Figure 6:**
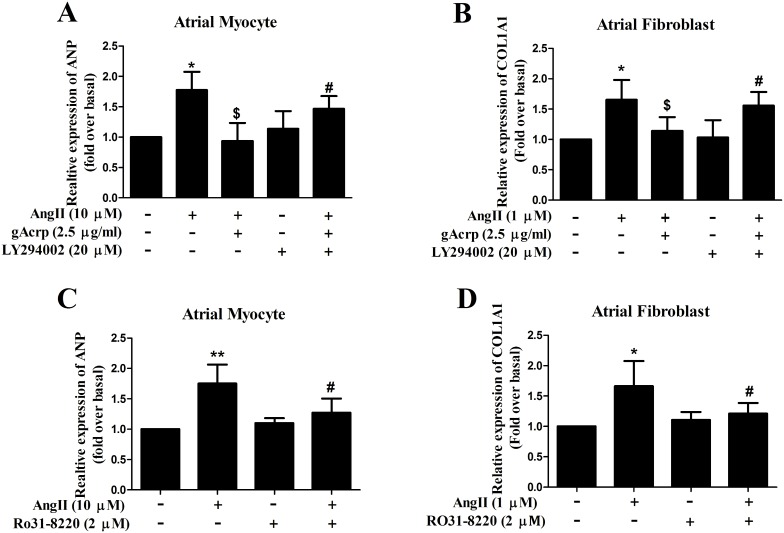
ANP and COL1A1 expression in atrial cells pretreated with 20 µM LY294002 or Ro31-8220. Atrial myocytes (A) and fibroblasts (B) were incubated with gAcrp (2.5 µg/ml), or pretreated with 20 µM LY294002 for 1 h and then incubated with AngII for 24 h. Data were expressed as mean ± SD of three independent experiments. *P<0.05 vs. blank control, ^$^ *P*<0.05 vs AngII infusion,^ #^ *P*<0.05 vs. AngII + gAcrp infusion. Atrial myocytes (C) and fibroblasts (D) were incubated with gAcrp (2.5 µg/ml), or pretreated with 2 µM Ro31-8200 for 1 h, and then incubated with AngII for 24 h. Data were expressed as mean ± SD of three independent experiments. *P<0.05; **P<0.01 vs. blank control, ^#^ *P*<0.05 vs. AngII + Ro31-8220.

AMPK is a critical metabolic regulator of adiponectin downstream signaling action. We previously demonstrated that activation of AMPK though adiponectin could protect atrial myocytes (data not shown). Moreover, the AMPK activator 5-aminoimidazole-4-caroxamide-1-β-d-ribofuranoside (AICAR, 1 nM) could simulate this effect (Figure. S2). In contrast, an AMPK inhibitor, compound C (10 µM), markedly inhibited this effect (Figure. S3). Thus, we investigated the underlying mechanism of how AMPK stimulates a series of metabolic pathways to ameliorate AngII-induced atrial hypertrophy and fibrosis. Western blot analysis detected AMPK and Akt activity after pretreatment with LY294002 (PI3K inhibitor, 20 µM). Fig. 5A showed that in atrial myocytes, treatment with gAcrp significantly increased AMPK phosphorylation, but there were no changes in the group with the addition of LY294002 (*lane 3 and 4*). However, pretreatment of cells with LY294002 prominently reduced the phosphorylation of Akt (*lane 4*). Atrial fibroblasts displayed the same phenomena (Fig. 5B). Together, these data indicated that the activation of Akt by gAcrp occurred through a pathway that required the activation of PI3K in atrial cells. Based on this signaling axis, atrial myocytes and fibroblasts were pretreated with LY294002 for 60 min and then treated with gAcrp (2 µg/ml). After incubation with AngII for 24 h, ANP and COL1A1 mRNA expression was analyzed by qPCR. As seen in Fig. 6A and 6B, acute treatment with LY294002 (*lane 5*, *p<*0.05) attenuated gAcrp protection against atrial hypertrophy and fibrosis. However, Ro31-8220, an Akt activator, exerted its function by inhibiting AngII-induced atrial hypertrophy and fibrosis (Fig. 6C and 6D *lane 4*, *p*<0.05).

### Globular adiponectin attenuated AngII-induced NF-κB activation and translocation in atrial myocytes via AMPK activation

**Figure 7:**
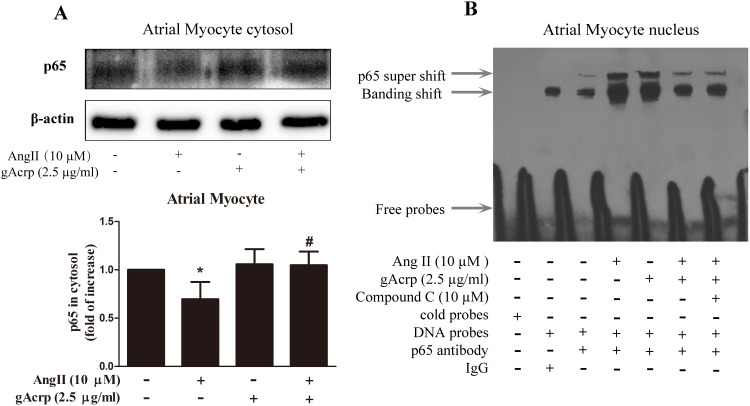
Effect of gAcrp on NF-κB translocation in atrial myocytes. (A) Atrial myocytes were pretreated with or without gAcrp (2.5 µg/ml) for 1 h and then stimulated AngII (10 µM) for 1 h. Cytosol proteins were extracted and immunblotted with anti-p65 antibody. Anti-β-actin antibody was used as control. Data were expressed as mean ± SD of three independent experiments. *P<0.05 vs. blank control, ^#^ *P*<0.05 vs. AngII infusion. (B) Atrial myocytes were pretreated with compound C (10 µM, *lane 7*) or gAcrp (2.5 µg/ml, *lane 6*), and then incubated with AngII for 60 min (*lanes 4*, *6* and *7*). Lanes 1 and 2 indicated that nonspecific probes and control IgG were regarded as negative controls. Lanes 3–7 used anti-p65 antibody to certify that all samples in the membrane are significant.

NF-κB activation has been reported to be essential for hypertrophic growth of atrial myocytes during translocation stimulated by the hypertrophic agonist AngII. Nuclear localization of NF-κB response to AngII was observed (Fig. 7) but globular adiponectin was found to abrogate this response. As shown in Fig. 7A, NF-κB p65 expression decreased in the cytosol (*lane 2*, *p*<0.05) as mediated by AngII. Notably, the decrease in cytosol was significantly reversed by the stimulation of gAcrp, whereas gAcrp alone had no significant effect on atrial myocyte basal NF-κB p65 expression (*lane 4*, *p*<0.05 and *lane 3*, *p*>0.05). Subsequently, we investigated the DNA p65 interaction to confirm that gAcrp inhibited NF-κB translocation in hypertrophic atrial myocytes induced by AngII. The EMSA results shown in Fig. 7B indicate that the DNA binding ability of NF-κB p65 was prominently increased (*lane 4*) by AngII but was suppressed by pretreatment with gAcrp (*lane 6*). Moreover, with pretreatment with compound C (10 µM, an AMPK inhibitor), a larger band shift was observed (*lane 7*). Anti-NF-κB p65 antibody supershift assays demonstrate that that all nuclear extracts specifically contained this transcription factor (*lanes 3–7*).

## Discussion

**Figure 8:**
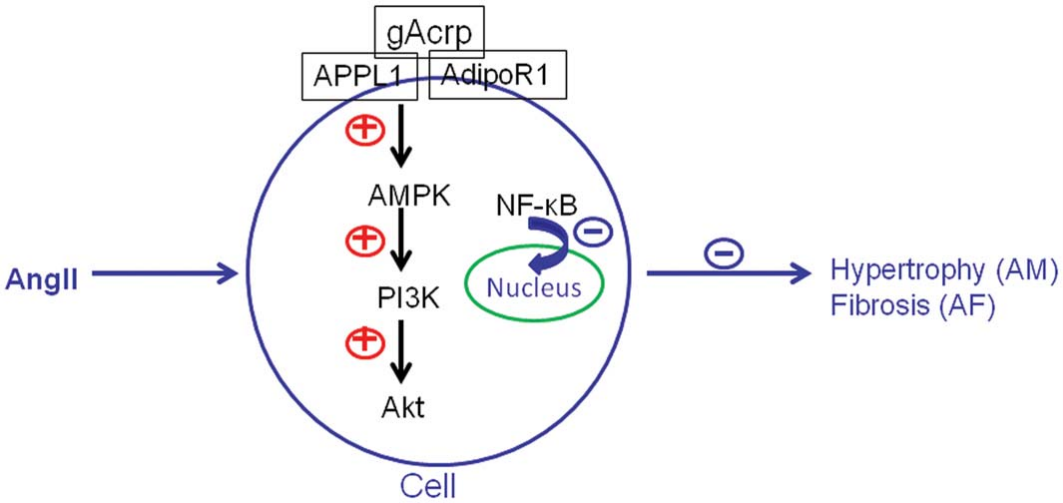
Schematic model of the proposed effects of gAcrp on Ang II-induced atrial hypertrophy and fibrosis. Treatment of AngII induces hypertrophy and fibrosis in atrial cells. gAcrp suppresses AngII-induced hypertrophy and fibrosis in atrial cells through activation of AMPK and downstream signaling pathways. The inhibitory effect of gAcrp on AngII-induced atrial hypertrophy and fibrosis is mediated through AdipoR1- and APPL1- dependent mechanisms.

Here we present new data showing that incubation of gAcrp disrupts AngII-induced atrial hypertrophy and fibrosis in the neonatal rat atrial cells via multiple signaling pathways (see schema in Fig. 8). We investigated the roles of AdipoR1 and APPL1 in the gAcrp-induced signaling pathway that leaded to protective effects against atrial hypertrophy and fibrosis in neonatal rat atrial myocytes and fibroblasts. Our results demonstrated that downregulation of AdipoR1 or APPL1 caused a prominent attenuation of gAcrp-induced AMPK activation and related downstream signaling pathways. Furthermore, we found that gAcrp, along with AMPK/PI3K/Akt, activated signaling, and accelerated NF-κB p65 translocation from the cytosol to the nucleus.

It is well known that plasma adiponectin is negatively regulated by obesity [17]. Adiponectin is the most abundant adipose tissue-derived hormone. Adiponectin exists in two multimers, full-length adiponectin and globular adiponectin [8]. In the present study, we concentrated on globular adiponectin, which has been demonstrated to produce multiple cardioprotective effects, including anti-hypertrophic, anti-fibrotic and anti-ischemia-reperfusion injury effects [10] because this form has a higher affinity for myocytes than full-length adiponectin [18]. Cloning of adiponectin receptors has facilitated the understanding of the molecular mechanisms of adiponectin action [13]. The suppression of AdipoR1 expression largely reduced gAcrp binding with it in myocytes [13].

It has been demonstrated that binding of the docking protein APPL1 with AdipoR1 interacts with the adiponectin signaling axis, and mediates metabolic effects in a variety of cell types [19], [20]. APPL1 also acts as a critical regulator of the crosstalk between adiponectin signaling and insulin signaling pathways [21]. Here, we demonstrated, for the first time, in primary neonatal rat atrial myocytes and fibroblasts that APPL1 regulates gAcrp action, promoting the expression of ANP and COL1A1 in hypertrophic atria. APPL1 acts as a vital mediator involved in adiponectin-induced AMPK, PI3K activation, phosphorylation of p38 MAPK and Akt [19], [21], [22]. In accordance with this finding, our proteomics-based analysis also clarified the interaction between APPL1 and the aforementioned signaling pathways. On the basis of these results, we can affirm that AMPK activation by gAcrp prevented AngII-induced hypertrophy and fibrillation.

Several pieces of evidence suggest that exogenous norepinephrine, endothelin-1 (ET-1), and AngII can trigger the pathogenesis of hypertrophic cultured cardiomyocytes and ventricular remodeling [23], [24]. Angiotensin has 7 subtypes with vasodilator property. AngII produces important cell signaling cascades and related effects on physiology and pathology in the cardiovascular system [25]. For example, through the G protein-dependent pathway, AngII activates serine/threonine kinases including ERK1/2, p38 MAPK, and JNK, which are responsible for cell growth and hypertrophy. Moreover, activation of JAK/STAT pathway is involved with nonreceptor tyrosine kinases by AngII [25]. A previous study demonstrated tyrosine phosphorylation of STAT3 by AngII in a dose-dependent and time-dependent manner in hypertrophic atrial myocytes and fibroblasts [5]. It is reasonable to speculate that ANP and COL1A1 could be regulated by AngII in a dose-dependent manner. Our results indicate significant increase of ANP and COL1A1 expression in the middle of the AngII concentration gradient (10 µM for ANP, and 1 µM for COL1A1) in neonatal rat atrial tissue.

Until recently, multiple gene regulating methods were used to modulate the expression of APPL1 and AdipoR1, the key adaptor protein of adiponectin and its downstream signaling pathway in cell metabolism [26]. Using transient transfection with APPL1 and AdipoR1 target siRNA, we found that both ANP and COL1A1 expressions were significantly inhibited, whereas the non-silencing siRNA did not produce similar effect. Our finding suggests that the inhibition of APPL1 and AdipoR1 expression could attenuate AMPK phosphorylation activated by gAcrp.

To further explore another possible mechanism of cardioprotection mediated by adiponectin, we attempted to investigate Akt and the major, but not only, upstream kinase PI3K in atrial cells. Adiponectin induced PI3K activation and adenoviral transduction of dominant-negative PI3K, and previous research verified that PI3K recovered Akt levels increased by adiponectin in endothelial cells [27]. The selective PI3K inhibitor LY294002 could blunt adiponectin-induced AMPK activation in endothelial cells [28]. Conversely, our results indicated that LY294002 alone activated AMPK instead of abrogating phosphorylation of AMPK. This discrepancy may be due to the different experimental methods, cell culture conditions *in* *vitro*, and animal species used. The decreased phosphorylation level of Akt could not be recovered with LY294002. These results again emphasize that the signaling axis PI3K/Akt was independent of AMPK in the atrial cells.

A number of downstream signaling pathways involved in adiponectin action have been revealed in recent years. AMPK is a serine/threonine protein kinase. Activation of AMPK by adiponectin can inhibit extracellular signal-regulated kinase (ERK) activity and a-adrenergic receptor norepinephrine-induced hypertrophy in neonatal cardiac ventricular myocytes [29]. Another pathway of Akt (protein kinase B) exerts a central role, involving the phosphatidylinositol 3-kinase (PI3K) pathway, in insulin-mediated glucose uptake. The process of the enhancement of insulin action on glucose uptake and phosphorylation of Akt was sensitized by adiponectin in the rat skeletal muscle L6 cell line [30]. Nuclear factor κB (NF-κB), is a key transcription factor that modulates the processes of inflammation, immune response and cell proliferation. Many studies have recently demonstrated that activation of NF-κB may be involved in cardiac structural remodeling [31]. In addition, the activation of NF-κB by AngII-induced cardiac hypertrophy was attenuated by adiponectin via a signaling transduction pathway that involves AMPK [12].

Akt plays a central role in cell signaling pathways of metabolism, proliferation and apoptosis. By binding to its receptors, gAcrp facilitates Akt phosphorylation in 3 myocytes cell lines [30]. Based on this finding, we hypothesize the selective Akt activator Ro31-8220 may exert the same effect as gAcrp in atrial myocytes and fibroblasts. Therefore, our data raises the intriguing possibility that the activation of Akt can mimic gAcrp action in playing a protective role in atrial cells while maintaining normal cardiac efficiency. Conversely, the simple inhibition of Akt cannot prevent the cardioprotective effect of gAcrp. Therefore, the PI3K/Akt signaling pathway is not the only pathway that can account for the protective role of gAcrp in hypertrophic atrial cells. The PI3K/Akt signaling pathway could be one of the most important signal transduction pathways that regulate atrium hypertrophy and fibrosis.

Previous studies have shown that adiponectin protects against myocardial hypertrophy via inhibition of NF-κB translocation, which is induced by AngII in ventricular cardiomyocytes [12]. In addition, the attenuation of NF-κB activation through the phosphorylation of AMPK plays a promotional role in vascular endothelial cells [32]. Our data clearly suggest that AngII can potently increase the protein expression of NF-κB p65 in nuclear extracts but can only slightly increase NF-κB p65 expression in cytoplasmic extracts. Consistent with the finding, strongly restrained AMPK activation can promote NF-κB p65 nuclear transfer, which reverses the signaling cascade activated by gAcrp.

Cardiomyocyte dysfunction leads to AF, which is a pathological condition closely related to obesity and diabetes. As a major adipocyte-secreted adipokine that is present abundantly in circulation, adiponectin has an important role in maintaining vascular homoeostasis, sustaining myocardial diastolic normalization [33] and suppressing atherosclerosis [20]. Nevertheless, the cellular mechanism underlying these protective effects of adiponectin does not account for its systemic effect. In this study, we further investigated the signaling cascade with adiponectin-triggered AMPK and PI3K pathways in atrial myocytes and fibroblasts. The present study focused on the *in* *vitro* model of hypertrophy and fibrosis using cultured cells. The *in* *vivo* effect of adiponectin and analysis of dysfunctional atrium tissue need to be further investigated.

## Conclusions

In conclusion, the current study provides novel supporting evidence that multiple signaling pathways may involve in the cardioprotective effect of adiponectin in neonatal rat hypertrophic atrial myocytes or fibrotic atrial fibroblasts, as induced by AngII. AMPK and PI3K participate the signal transduction through AdipoR1, and the anchoring protein APPL1 is involved in both the gAcrp/AMPK/NF-κB and gAcrp/AMPK/PI3K/Akt signaling axis as part of effective pathways for protection against atrium hypertrophy and fibrillation. Moreover, this report may provide preliminary evidence on the mechanisms of AngII-induced atrial structural remodeling in AF and atrial metabolic disorder.

## Supporting Information

Figure S1 **Compound C partially inhibits the cardioprotection mediated by gAcrp.** Atrial myocytes were incubated with gAcrp (2.5 µg/ml), or pretreated with compound C (AMPK inhibitor, 10 µM) for 1 h and then incubated with AngII for 24 h. Data were expressed as mean ± SD of three independent experiments. **P<0.01 vs. blank control, ^$$^ *P*<0.01 vs AngII infusion, ^##^ *P*<0.01 vs. AngII+ gAcrp infusion.
(DOC)

Figure S2 **AICAR inhibits hypertrophy of atrial myocytes induced by AngII.** Atrial myocytes were pretreated with specific activator of AMPK (AICAR, 1 nM) for 1 h, and then incubated with AngII (10 µM) in the presence of AICAR for 24 h. Data were expressed as mean ± SD of three independent experiments. **P<0.01 vs. blank control, ^##^ *P*<0.01 vs AngII infusion.
(DOC)

Figure S3 **Photomicrographs (X100) of cultured atrial myocytes and atrial fibroblasts incubated 72** **h after seeding on 6-wells plate.** (A) Atrial myocytes beating rate approaching 110 times per minute. (B) Atrial fibroblasts.
(DOC)
