# Supplementary material for: AdipoR1/APPL1 Potentiates the Protective Effects of Globular Adiponectin on Angiotensin II-Induced Cardiac Hypertrophy and Fibrosis in Neonatal Rat Atrial Myocytes and Fibroblasts
Source: PLoS One. 2014 Aug 6;9(8):e103793. doi: 10.1371/journal.pone.0103793 (PMC4123880; doi:10.1371/journal.pone.0103793)
Supplement: Figure S1 — Compound C partially inhibits the cardioprotection mediated by gAcrp. Atrial myocytes were incubated with gAcrp (2.5 µg/ml), or pretreated with compound C (AMPK inhibitor, 10 µM) for 1 h and then incubated with AngII for 24 h. Data were expressed as mean ± SD of three independent experiments. **P<0.01 vs. blank control, $$ P<0.01 vs AngII infusion, ## P<0.01 vs. AngII+ gAcrp infusion. (DOC) [file pone.0103793.s001.doc]

A


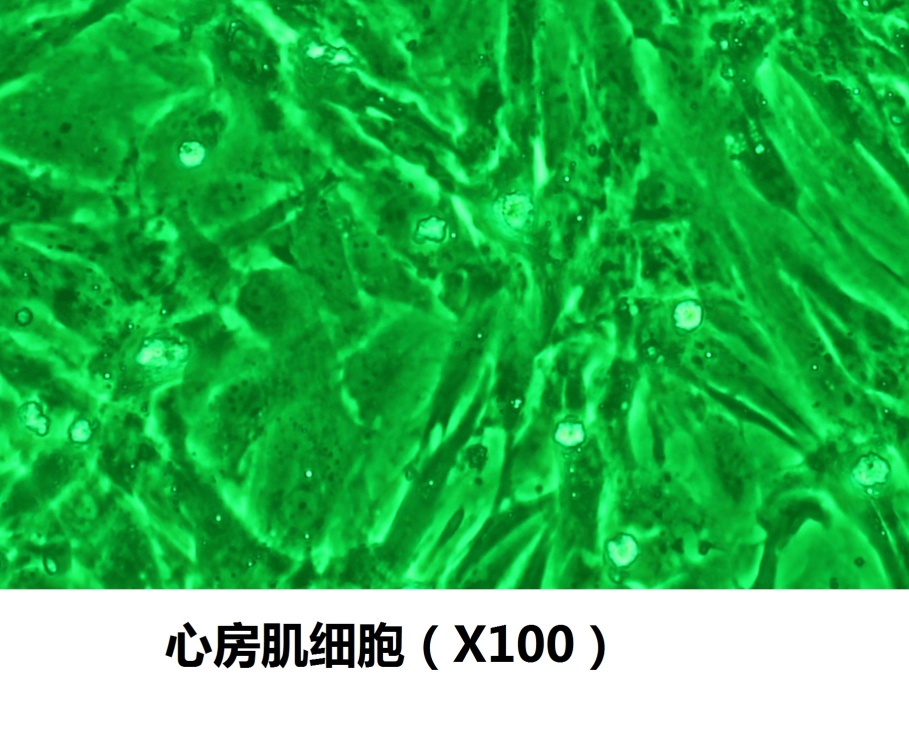


B


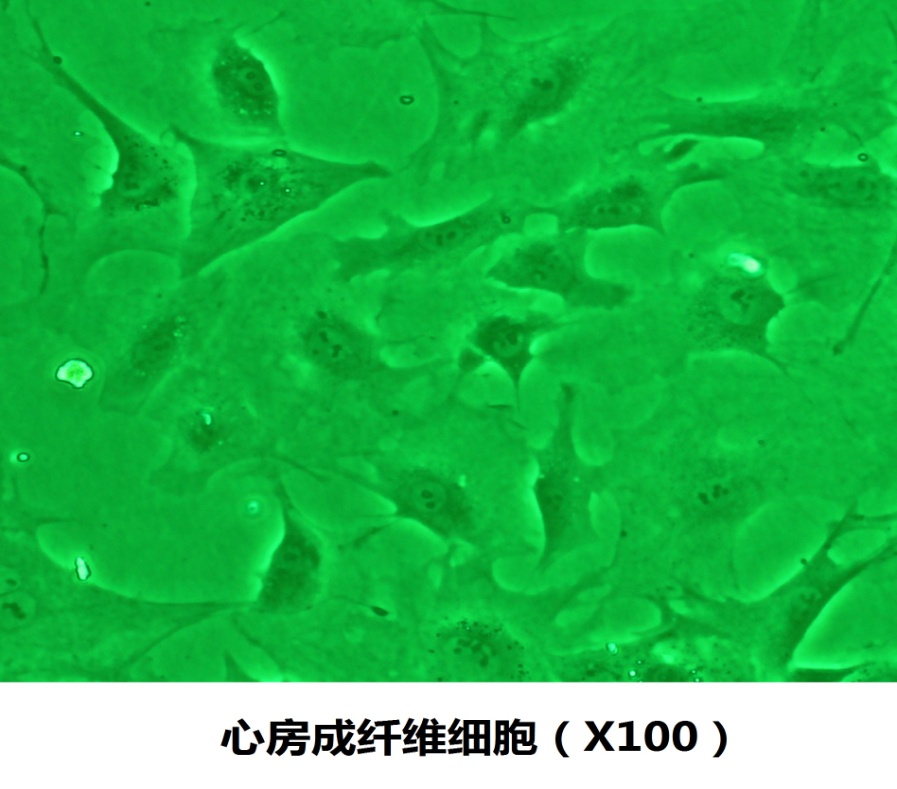


Figure. S1 **Photomicrographs (X100) of cultured atrial myocytes and atrial fibroblasts incubated 72 h after seeding on 6-wells plate**. (A) Atrial myocytes beating rate approaching 110 times per minute. (B) Atrial fibroblasts.
