# Supplementary material for: AdipoR1/APPL1 Potentiates the Protective Effects of Globular Adiponectin on Angiotensin II-Induced Cardiac Hypertrophy and Fibrosis in Neonatal Rat Atrial Myocytes and Fibroblasts
Source: PLoS One. 2014 Aug 6;9(8):e103793. doi: 10.1371/journal.pone.0103793 (PMC4123880; doi:10.1371/journal.pone.0103793)
Supplement: Figure S2 — AICAR inhibits hypertrophy of atrial myocytes induced by AngII. Atrial myocytes were pretreated with specific activator of AMPK (AICAR, 1 nM) for 1 h, and then incubated with AngII (10 µM) in the presence of AICAR for 24 h. Data were expressed as mean ± SD of three independent experiments. **P<0.01 vs. blank control, ## P<0.01 vs AngII infusion. (DOC) [file pone.0103793.s002.doc]

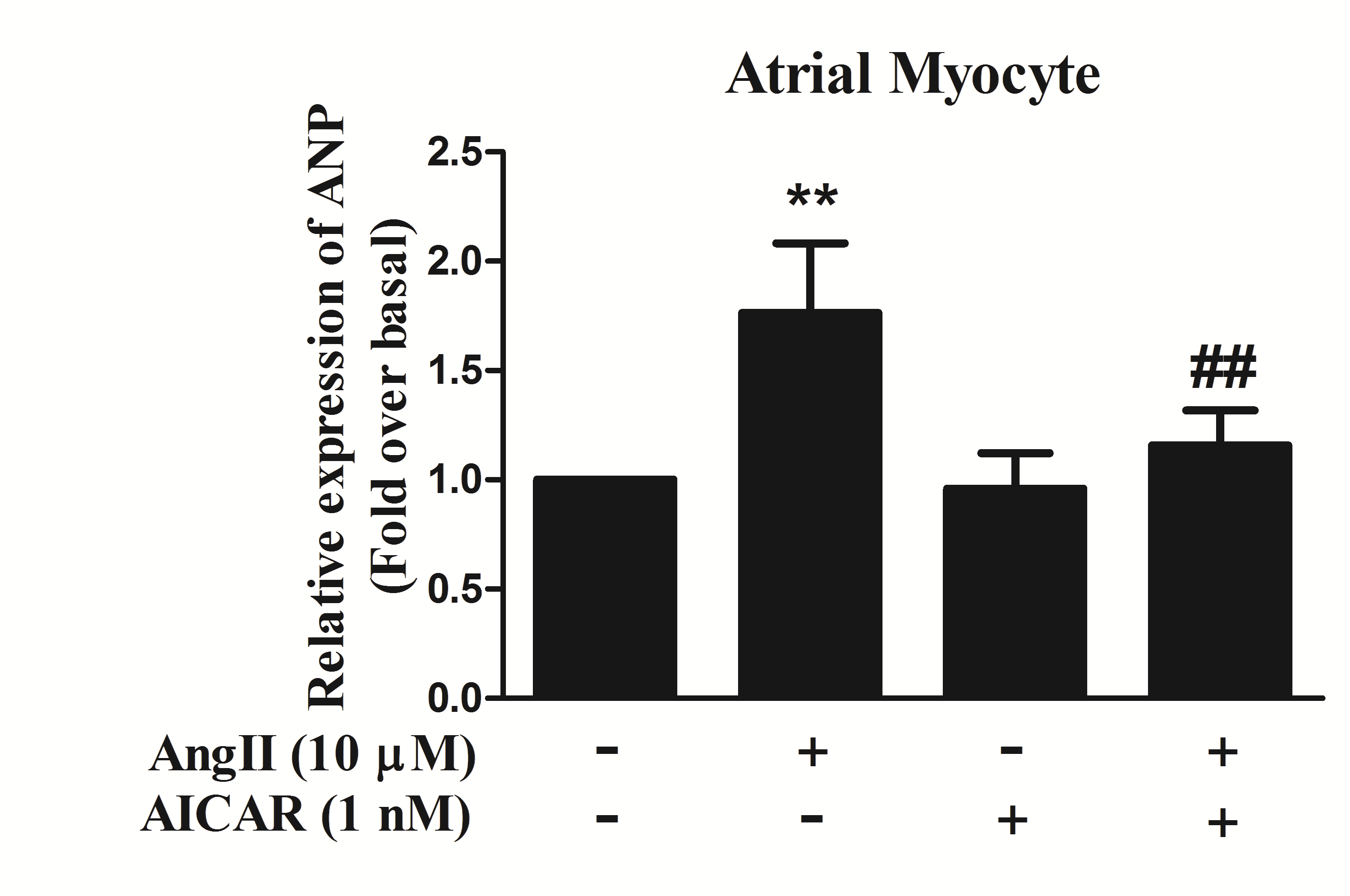


Figure. S2 **AICAR inhibit hypertrophy of atrial myocytes induced by AngII**. Atrial myocytes were pretreated with specific activator of AMPK (AICAR, 1 nM) for 1 h, and then together with AngII (10µM) for 24 h. Data were expressed as mean ± SD of three independent experiments. ^**^*P*<0.01 vs. blank control, ^##^*P*<0.01 vs AngII infusion.
