# Supplementary material for: AdipoR1/APPL1 Potentiates the Protective Effects of Globular Adiponectin on Angiotensin II-Induced Cardiac Hypertrophy and Fibrosis in Neonatal Rat Atrial Myocytes and Fibroblasts
Source: PLoS One. 2014 Aug 6;9(8):e103793. doi: 10.1371/journal.pone.0103793 (PMC4123880; doi:10.1371/journal.pone.0103793)
Supplement: Figure S3 — Photomicrographs (X100) of cultured atrial myocytes and atrial fibroblasts incubated 72 h after seeding on 6-wells plate. (A) Atrial myocytes beating rate approaching 110 times per minute. (B) Atrial fibroblasts. (DOC) [file pone.0103793.s003.doc]

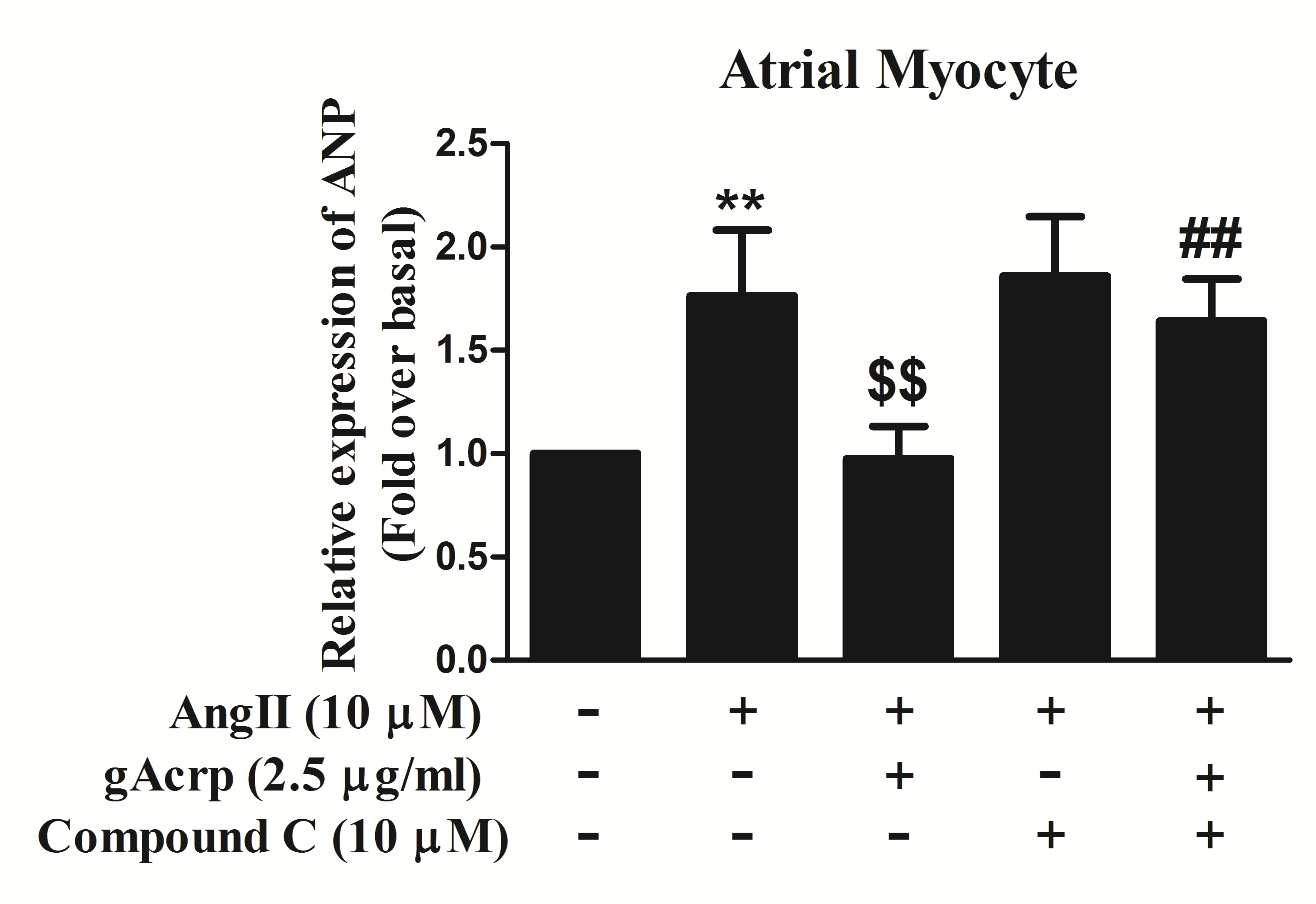


Figure. S3 **Compound C partially inhibit function of cardioprotection mediated by gAcrp.** Atrial myocytes were incubated with gAcrp (2.5 µg/ml), or pretreated with compound C (AMPK inhibitor, 10 µM) for 1 h then mediated with AngII for 24 h. Data were expressed as mean ± SD of three independent experiments. ^**^*P*<0.01 vs. blank control, ^$$^*P*<0.01 vs AngII infusion, ^##^*P*<0.01 vs. AngII+ gAcrp infusion.
